# Sluggishness of Early-Stage Face Processing (N170) Is Correlated with Negative and General Psychiatric Symptoms in Schizophrenia

**DOI:** 10.3389/fnhum.2016.00615

**Published:** 2016-11-28

**Authors:** Yingjun Zheng, Haijing Li, Yuping Ning, Jianjuan Ren, Zhangying Wu, Rongcheng Huang, Guoming Luan, Tianfu Li, Taiyong Bi, Qian Wang, Shenglin She

**Affiliations:** ^1^Department of General Psychiatry, The Affiliated Brain Hospital of Guangzhou Medical University (Guangzhou Huiai Hospital)Guangzhou, China; ^2^Beijing Key Laboratory of Epilepsy, Epilepsy Center, Department of Functional Neurosurgery, Sanbo Brain Hospital, Capital Medical UniversityBeijing, China; ^3^Beijing Institute for Brain DisordersBeijing, China; ^4^Key Laboratory of Cognition and Personality (SWU), Ministry of EducationChongqing, China; ^5^Faculty of Psychology, Southwest UniversityChongqing, China

**Keywords:** schizophrenia, N170, face processing, schizophrenia symptoms, demyelination

## Abstract

Patients with schizophrenia consistently exhibit abnormalities in the N170 event-related potential (ERP) component evoked by images of faces. However, the relationship between these face-specific N170 abnormalities in patients with schizophrenia and the clinical characteristics of this disorder has not been elucidated. Here, ERP recordings were conducted for patients with schizophrenia and healthy controls. The amplitude and latency of the N170 component were recorded while participants passively viewed face and non-face (table) images to explore the correlation between face-specific processing and clinical characteristics in schizophrenia. The results provided evidence for a face-specific N170 latency delay in patients with schizophrenia. The N170 latency in patients with schizophrenia was significantly longer than that in healthy controls when images of faces were presented in both upright and inverted orientations. Importantly, the face-related N170 latencies of the left temporo-occipital electrodes (P7 and PO7) were positively correlated with both negative and general psychiatric symptoms in these patients. The N170 amplitudes were weaker in patients than in controls for inverted images of both faces and non-faces (tables), with a left-hemisphere dominance. The face inversion effect (FIE), meaning the difference in N170 amplitude between upright and inverted faces, was absent in patients with schizophrenia, suggesting an abnormality of holistic face processing. Together, these results revealed a marked symptom-relevant neural delay associated with face-specific processing in patients with schizophrenia, providing additional evidence to support the demyelination hypothesis of schizophrenia.

## Introduction

Schizophrenia is a psychiatric disorder that is marked by positive symptoms, negative symptoms, cognitive deficits, motor abnormalities and social dysfunction (for example see Onitsuka et al., 2013). One consistent cognitive dysfunction in people diagnosed with schizophrenia involves non-emotional face processing (for reviews see Darke et al., 2013; Onitsuka et al., 2013; Watson, 2013; Bortolon et al., 2015; McCleery et al., 2015) both at behavioral (Chen, 2011; Maher et al., 2015, 2016) and physiological levels (Herrmann et al., 2004; Onitsuka et al., 2006; Tsunoda et al., 2012; Maher et al., 2016).

Event-related potentials (ERPs) are cortical electrophysiological responses to the presentation of a stimulus. A negativity that peaks at around 170 ms (N170) after stimulus onset, which is markedly enhanced by faces rather than by objects (Bentin et al., 1996), has been localized mainly to the fusiform face area (Rossion et al., 2003). Face-specific N170 potentials are significantly reduced in people with schizophrenia (Herrmann et al., 2004; Onitsuka et al., 2006; Tsunoda et al., 2012; Maher et al., 2016), suggesting that they have basic perceptual deficits in face processing.

Theoretically, individuals process face holistically, which has been shown by the face inversion effect (FIE) in which face discrimination performance for inverted faces is reduced compared with that for upright faces (Yin, 1969, 1970; Bauser et al., 2012). Previous studies have shown that the behavioral FIE is reduced or even absent in people with schizophrenia (Shin et al., 2008; Kim et al., 2010; Bauser et al., 2012; Megreya, 2016), suggesting a configural processing dysfunction. The FIE may be reflected at the electrophysiological level, with the N170 potential delayed and enhanced by an inverted face relative to an upright face in normal controls (McCarthy et al., 1999; Taylor et al., 2001; Itier and Taylor, 2002, 2004). To date, only one study has suggested that the FIE of N170 is significantly reduced in individuals with schizophrenia (Tsunoda et al., 2012). Although many studies have investigated the behavioral FIE deficit in people with schizophrenia, the FIE of the N170 in those with schizophrenia and its correlation with schizophrenia symptoms are unexplored.

The clinical symptoms of schizophrenia include positive symptoms, such as hallucinations and delusions, and negative symptoms, such as apathy and avolition. Whether there is a correlation between face-processing deficits and clinical symptoms in patients with schizophrenia remains controversial. Some behavior studies have shown that neither positive nor negative symptoms are accompanied by face discrimination performance (Addington and Addington, 1998; Baudouin et al., 2002; Caharel et al., 2007), whereas other studies find significant correlations between negative symptoms and face recognition performance (Sachs et al., 2004; Martin et al., 2005; Norton et al., 2009; Chen et al., 2012), and one study found significant correlations between both positive and negative symptoms and face identity performance (Chen et al., 2009). Although many studies have examined the correlation between face-processing behavioral indices and schizophrenic symptoms, few have explored the correlation between face-processing electrophysiological indices and schizophrenia symptoms. Thus, this correlation was assessed in the current study.

This study used a two (face, non-face) by two (upright, inverted) image design and electroencephalogram (EEG) recording to accomplish three goals. First, both the amplitude and latency of the N170 potential were measured in patients with schizophrenia and in healthy controls. Here, we tested the hypothesis that the face-specific N170 in patients with schizophrenia has smaller amplitude and enhanced delayed latency compared with those in healthy controls. Second, we measured the FIE of the N170 in these two populations to test the hypothesis that the FIE of N170 is absent in patients with schizophrenia. Finally, we assessed the correlation between the schizophrenia symptom scores and the N170 indices (including N170 amplitude, latency and FIE). Our overall aim was to determine whether N170 can be used an electrophysiological marker that objectively reflects the severity of schizophrenia.

## Materials and Methods

### Participants

This study was approved by the Institutional Review Board of Guangzhou Brain Hospital. 24 patients with schizophrenia (12 women, mean age 32.3 ± 11.2 years old) and 24 age-matched healthy controls (12 women, mean age 32.9 ± 11.5 years old) participated in this study. All participants reported normal or corrected-to-normal vision. Each patient was diagnosed with schizophrenia according to the Diagnostic and Statistical Manual of Mental Disorders, Fourth Edition. Their psychiatric symptoms were evaluated by a trained psychiatrist or psychologist based on the Positive and Negative Syndrome Scale (Kay et al., 1987). The Personal and Social Performance scale, an acceptable, quick, and valid measure (Morosini et al., 2000), was conducted to assess participants’ social functioning. No patient had a history of severe medical or severe neurological disorders. The demographic and descriptive characteristics of the participants are shown in Supplementary Table S1. No healthy volunteer reported a history of major psychiatric disorder or major physical illness or use of medication known to affect the central nervous system. The exclusion criteria for both groups included clear abnormality of MRI results, neurological illness, traumatic brain injury, substance abuse, or addiction. All participants received financial compensation and provided written informed consent for their participation and all the methods were performed in accordance with the clinical-experimental guidelines of human experiment approved by Institutional Review Board of Guangzhou Brain Hospital. No vulnerable populations were involved in this study.

### Stimuli and Procedure

**Figure 1:**
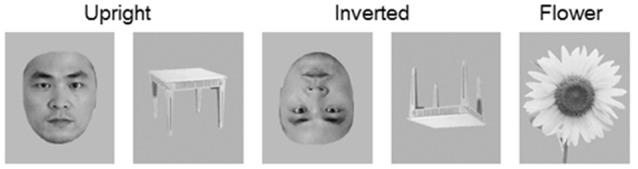
**Examples of target (flowers) and non-target stimuli (faces and tables) in upright and inverted orientations.** Only the non-target stimulus-evoked event-related potential (ERP) were analyzed.

The stimuli were grayscale photographs (10.58 cm × 12.70 cm) of 60 unfamiliar young faces, 60 tables and 62 flowers. Half of the faces were male, and all faces were presented without hair atop the head (facial hair was included), glasses, or other accessories (Figure 1). These stimuli were used to form five stimulus conditions: (a) upright faces; (b) upright tables; (c) inverted faces; (d) inverted tables; and (e) targets (flowers). All images were equated for luminance and root mean square contrast (calculations excluded the gray background) using Adobe Photoshop^1^ (Gao et al., 2009). The stimuli were presented at fixation, and seen from a distance of 1.2 m, they occupied a visual angle of 5.051° × 6.061°.

Participants were tested in a dimly lit room during the experiment. They sat in a comfortable chair and were instructed to maintain a mental count of the images of flowers they saw on the screen and to ignore all other stimuli. This procedure has been used in research examining the N170 component to assign similar task relevance to face and non-face stimuli (e.g., Carmel and Bentin, 2002; Gao et al., 2009). The 302 stimuli were presented one at a time in two blocks: one block comprised 20 upright faces, 20 inverted faces, 20 upright tables, 20 inverted tables and 20 flowers (targets); and the other block comprised 40 upright faces, 40 inverted faces, 40 upright tables, 40 inverted tables and 42 flowers (targets). The order of trials within each block was randomized. Stimulus exposure time was 250 ms and was separated by an inter-trial interval randomized from 750 ms to 1250 ms. There was a 1-min break between blocks.

### Electrophysiological Recording

The EEG was recorded continuously by a set of 16 Ag/AgCl electrodes placed according to the 10/20 system. The EEG recording sites were: F3, Fz, F4, C3, Cz, C4, P7, P3, Pz, P4, P8, PO7, PO8, O1, Oz and O2. An electrooculogram (EOG) was recorded to monitor eye movements and blinks via electrodes placed on bilateral external canthi and the left infraorbital and supraorbital areas. EEG and EOG signals were sampled at 1000 Hz, with a 0.1–100 Hz bandpass filter using a NuAmps digital amplifier system (Neuroscan Labs, El Paso, TX, USA). The tip of the nose was used as a reference during recording, and determination of the approximate zero reference using the reference electrode standardization technique (REST^2^) was conducted off-line (Yao, 2001; Tian and Yao, 2013). Electrode impedances were kept below 5 kΩ.

### Data and Statistical Analyses

The pre-processing of the electrophysiological data was conducted using the EEGLAB toolbox (Delorme and Makeig, 2004) in the MATLAB environment. The long-term EEG of each electrode was first bandpass filtered (1–100 Hz) and then segmented into epochs from −100 ms to 400 ms around the onset. The baseline correction was conducted using a time window of −100 ms to 0 ms. Epochs that contained more than ±1 mV potential were rejected as artifacts. The remaining epochs were averaged to obtain an ERP for each electrode node and then low-pass filtered at 15 Hz.

Statistical analysis was performed with IBM SPSS Statistics 20 software (SPSS Inc., Chicago, IL, USA). Analyses of variance (ANOVAs), *t*-tests and Spearman correlations were conducted. The *p* values were corrected by Bonferroni adjustment for multiple comparisons. The null-hypothesis rejection level was set at 0.05.

## Results

Target (flower) monitoring was equally great in both groups: 98.3% for patients with schizophrenia and 99.5% for healthy controls (*p* > 0.05, independent samples *t*-test). Non-target stimuli (i.e., faces and tables) elicited clear P1 and N170 components, with significant temporo-occipital distribution in both the patient and healthy control groups (Supplementary Figure S1). Only the N170 component was further analyzed.

After comparing the REST and average reference (AR) results (Supplementary Tables S3, S5), we found that REST gave more significant results around the temporo-occipital area (Supplementary Figures S1, S2). Thus, the REST results are presented here, and the AR- and REST-based results are compared in the supplementary materials.

### N170 Latency and Amplitude

**Figure 2:**
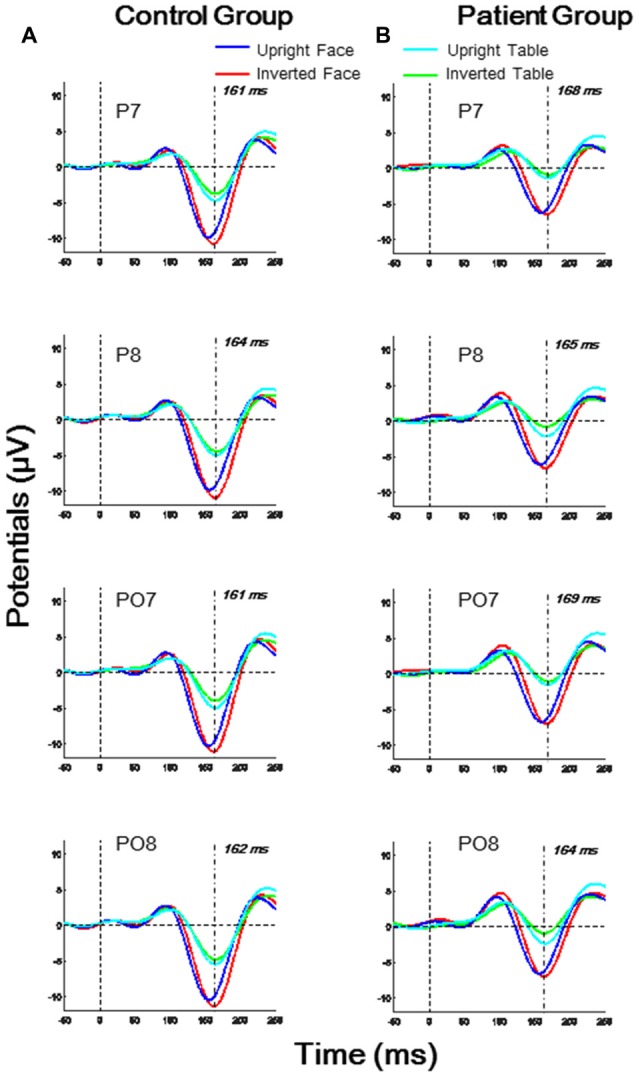
**Temporo-occipital ERP waveforms (electrodes P7, P8, PO7, PO8) evoked using four stimulus conditions (upright and inverted images of faces and tables) in both control and patient groups. (A)** Control group; **(B)** Patient group.

As shown in Figure 2, the N170 latencies and amplitudes differed for the four experimental stimulus conditions in each group. Furthermore, the N170 waveform was generally lower in the patient group than in the control group. To evaluate the group and stimulus effects on the N170 potential, we conducted a three-way, mixed-measures ANOVAs for latencies and amplitudes: 2 (group effect: patient, control) × 2 (Face effect: face, table) × 2 (inversion effect: upright, inverted).

For the N170 latency analysis, significant group effects were found in O1, P7 and PO7 electrodes (all *p* < 0.05) and significant inversion effects were found in P7, P8, and PO8 electrodes (all *p* < 0.05). A significant effect of face was found in P8 and PO8 (both *p* < 0.01). A significant interaction for face × group was found in P8 (*F*_(1,46)_ = 4.674, *p* = 0.036), and a significant interaction of face × inversion was found in PO7 (*F*_(1,46)_ = 6.918, *p* = 0.012; details shown in Supplementary Table S2).

For the N170 amplitude analysis, a significant group effect was found only in P7 and PO7 electrodes (both *p* < 0.05), and significant effect of face was found in O1, O2, P7, P8, PO7, PO8 and Oz electrodes (all *p* < 0.01). No significant inversion effect was found. A significant interaction of face × inversion was found in P7 and PO7 (both *p* < 0.05). No other significant interaction was observed in any electrode (details shown in Supplementary Table S4).

### N170 Group Differences

**Figure 3:**
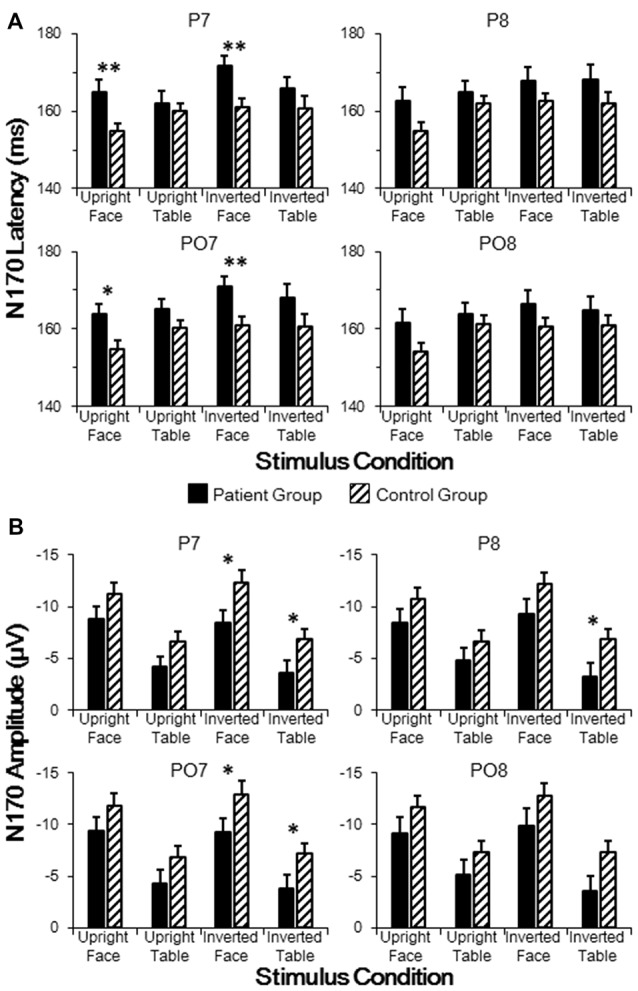
**Comparisons of N170 latencies (A) and amplitudes (B) in patient and control groups in temporo-occipital electrodes (P7, P8, PO7 and PO8).** Point lines: measurement of the peak latency when the face image was oriented upright. *p < 0.05, **p < 0.01.

As stated above, group effects for both N170 latency and amplitude were observed around the P7 electrode. To further analyze across group differences, we conducted independent samples *t*-tests between the patient and control groups for electrodes P7, P8, PO7 and PO8 for each of the four stimulus conditions. As shown in Figure 3A, the N170 peak latencies in patients were significantly longer than those in controls for both upright faces (P7: *t*_[46]_ = 3.036, *p* = 0.004; PO7: *t*_[46]_ = 2.861, *p* = 0.006) and inverted faces (P7: *t*_[46]_ = 3.022, *p* = 0.004; PO7: *t*_[46]_ = 2.795, *p* = 0.008) in the left hemisphere, without any significance difference in the right hemisphere (all *p* > 0.05 for P8 and PO8). These results indicated sluggishness for the face-evoked N170 in patients with schizophrenia, with left hemisphere dominance.

As shown in Figure 3B, the N170 amplitude in patients was significantly smaller than that in controls for both inverted faces (P7: *t*_[46]_ = 2.432, *p* = 0.019; PO7: *t*_[46]_ = 2.223, *p* = 0.031) and inverted tables (P7: *t*_[46]_ = 2.159, *p* = 0.036; PO7: *t*_[46]_ = 2.025, *p* = 0.049) in the left hemisphere, while a significant difference in the right hemisphere was found only for inverted tables at P8 (*t*_[46]_ = 2.100, *p* = 0.041; all other comparisons *p* > 0.05 for P8 and PO8). These results indicated that inverted face-evoked N170 amplitudes were reduced in patients with schizophrenia.

The differences between the stimulus conditions in each group were assessed using *post hoc t*-tests. In the control group, the N170 latency evoked by upright faces was significantly delayed compared with that evoked by upright tables (P8: *p* = 0.035; PO7: *p* = 0.006; PO8: *p* = 0.004). However, in the patient group, the N170 latencies evoked by upright faces and upright tables were not significantly different (all *p* > 0.05). In both control and patient groups, the N170 latency evoked by inverted faces was significantly larger than that evoked by upright faces (P7: both *p* < 0.001; P8: both *p* < 0.001; PO7: both *p* < 0.001; PO8: both *p* < 0.001). No inversion effect was found for the table stimuli in either group (all *p* > 0.05). In both control and patient groups, the N170 amplitudes evoked by faces were significantly larger than those evoked by either upright or inverted tables (P7: all *p* < 0.001; P8: all *p* < 0.001; PO7: all *p* < 0.001; PO8: all *p* < 0.001). In both groups, the N170 amplitude showed no significant difference between upright and inverted tables (all *p* > 0.05).

### N170 Latency Correlated With Symptom Scores

Spearman correlation tests were conducted for the N170 latencies and amplitudes (P7, P8, PO7 and PO8) of individual participants in both groups against the three clinical symptom scores, namely positive, negative and general psychiatric symptom scores.

**Figure 4:**
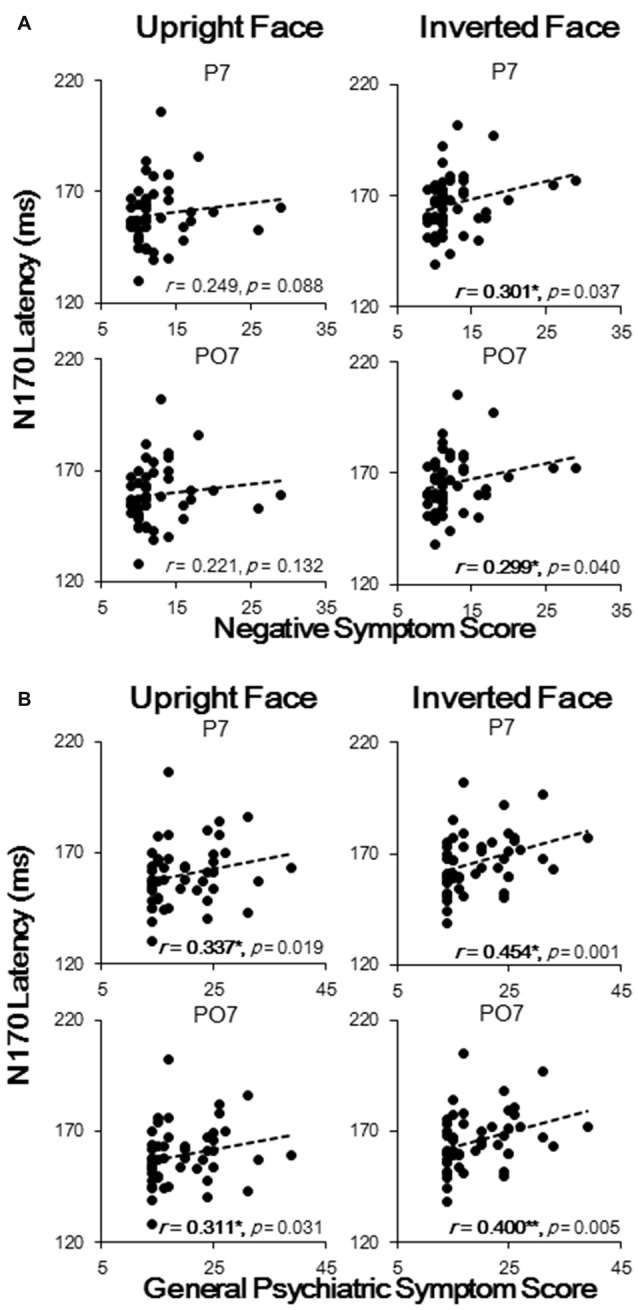
**Spearman correlation analysis results of N170 latencies with negative symptom scores (A)** and with general psychiatric symptom scores **(B)**. Each black dot represents an individual participant. *p < 0.05; **p < 0.01.

The results showed that N170 latencies were correlated with negative symptom and general psychiatric symptom scores, with no significant correlation found between N170 latencies and positive symptom scores. To be more specific, the upright face-evoked N170 latencies in P7 and PO7 were significantly and positively correlated with general psychiatric symptom scores (both *p* < 0.05; Figure 4B), and the inverted face-evoked N170 latencies in P7 and PO7 were significantly and positively correlated with both negative symptom scores (Figure 4A) and general psychiatric symptom scores (all *p* < 0.05; Figure 4B). Thus, significant correlations were found only for face stimuli, not for table stimuli. No significant correlation was found in P8 or PO8 between any symptom and stimulus condition. Similarly, no significant correlation was found between the N170 amplitude and any symptom score. These results indicated that N170 latency sluggishness may effectively predict clinical symptom scores at the level of the individual.

### Abnormal Face Inversion Effect

To evaluate whether upright and inverted faces were differentially processed in the patient and control groups, we calculated FIE scores for each participant, which was defined as N170 amplitudes evoked by upright faces minus those evoked by inverted faces. One-sample *t*-tests were used to determine whether the FIE score was significantly larger than 0. We found that the FIE scores in the control group were significantly larger than 0 (P7: *t*_[23]_ = 2.484, *p* = 0.021; P8: *t*_[23]_ = 3.679, *p* = 0.001; PO7: *t*_[23]_ = 2.232, *p* = 0.036; PO8: *t*_[23]_ = 2.753, *p* = 0.011), whereas the FIE scores in the patient group were not significantly different from 0 (Supplementary Figure S3). However, no significant correlation was found using Spearman correlation tests between FIE and symptom scores. These results demonstrated abnormality of holistic face processing in patients with schizophrenia.

## Discussion

### Face-Specific Deficits in Schizophrenia

The dysfunction of non-emotional face processing in patients with schizophrenia is controversial because such a deficit in these patients is not always specific to faces (for reviews see Darke et al., 2013; Onitsuka et al., 2013; Watson, 2013; Bortolon et al., 2015; McCleery et al., 2015). Although the face-specific area is mainly localized to the fusiform gyrus, neurophysiological studies have reported decreased activities (Desco et al., 2003; Silverstein et al., 2010) and reductions in the total neuron number (Dorph-Petersen et al., 2007) in the early visual cortex (V1–V3) in patients with schizophrenia. To extract the face-specific components for holistic processing, we used images of both upright and inverted faces together with non-face images to generate N170 ERPs in both patients with schizophrenia and healthy controls.

As shown in Figure 3, the N170 component was reduced and delayed in patients compared with that in healthy controls, which is consistent with the results of previous ERP studies (Herrmann et al., 2004; Onitsuka et al., 2006; Tsunoda et al., 2012). More specifically, in patients with schizophrenia, the N170 latencies were delayed only under face-evoked conditions (both upright and inverted). By contrast, the decrease in the N170 amplitudes showed no face selectivity. In addition, the face-evoked N170 latencies were positively correlated with negative symptom scores and general psychiatric symptom scores (Figure 4). No significant correlations were found for N170 latencies under non-face-evoked conditions, indicating that the face-specific N170 latencies could, to a certain extent, predict the severity of schizophrenia symptoms. Therefore, the face-specific N170 latency may be a useful neurophysiological index of some symptoms in patients with schizophrenia.

Neurophysiological function in schizophrenia is thought to be asymmetrical across hemispheres (Crow et al., 1989; Gruzelier, 1999). In this study, the face-specific N170 latency was delayed only in the left hemisphere of patients with schizophrenia. This hemisphere asymmetry is further supported by structural studies that have identified a smaller left fusiform gyrus gray matter volume in schizophrenic patients compared with those in healthy controls (McDonald et al., 2000; Lee et al., 2002). These results demonstrated a left hemisphere involvement of face-specific dysfunction in patients with schizophrenia.

### Face Inversion Effect Absence in Schizophrenia

A major hypothesis for the processing of global face configurations requires that additional areas be involved in the processed (Eimer, 2000a,b; Itier and Taylor, 2004), which is supported by an ERP dipole analysis study (Watanabe et al., 2003) and an ERP competition paradigm (Sadeh and Yovel, 2010). Moreover, an fMRI study revealed that inverted face processing recruits regions involved in face or object processing (Haxby et al., 1999). Rosburg et al. (2010) illustrated that the lateral occipital cortex contributes to the FIE of ERPs. In our study (shown in Supplementary Figure S2), significant increases in the N170 amplitude evoked by inverted faces compared with those by upright faces (i.e., the FIE) were observed only in healthy controls, not in patients with schizophrenia, which is consistent with the results of a previous study (Tsunoda et al., 2012). However, we found no significant correlation between the FIE and any clinical symptom. These results suggest that the face-selective cortex may be associated with the pathophysiology in schizophrenia. According to the study by Tsunoda et al. (2012), the FIE is absent in patients with schizophrenia, which might be related to social function. Thus, the abnormality of neural responses in areas related to holistic face processing may have a close relationship to social dysfunction but not to clinical symptoms.

### The Demyelination Hypothesis of Schizophrenia

In this study, face-specific N170 latency was delayed in the patient group, and this sluggishness was positively correlated with schizophrenia symptoms (Figures 3, 4). These findings lead to the hypothesis that a common neural mechanism underlies both clinical symptoms and face-processing defects in patients with schizophrenia.

One of the most popular perspectives accounting for the neuropathology observed in schizophrenia is the demyelination hypothesis (Chance et al., 1999; Foong et al., 2001; Hakak et al., 2001; Uranova et al., 2001; Davis et al., 2003; Flynn et al., 2003; Kubicki et al., 2005), which can explain the white matter damage (Okugawa et al., 2002; Federspiel et al., 2006; DeLisi, 2008; Friedman et al., 2008; Gasparotti et al., 2009), brain network synchrony reductions (Spencer et al., 2003, 2004; McIntosh et al., 2008; Uhlhaas et al., 2008; Peters et al., 2010; Uhlhaas and Singer, 2010), and genetic defects (Hakak et al., 2001; McCullumsmith et al., 2007) in schizophrenic patients with a simple myelin-related dysfunction mechanism.

Previous studies have shown that myelin-related defects are relevant to schizophrenia symptoms (Sanfilipo et al., 2000; Sigmundsson et al., 2001), and damage to myelin can result in conduction delays in neural discharge (Whitford et al., 2012). For instance, the visual-evoked P1 components in patients with multiple sclerosis are delayed about 20 ms because of damage to the optic nerve myelin (Cowan et al., 1984; Brusa et al., 2001). In the current study, the face-specific N170 latency was delayed in the patient group compared with that in healthy controls, and this sluggishness was positively correlated with negative symptoms and general psychiatric symptoms, providing additional evidence supporting the demyelination hypothesis of schizophrenia.

### Limitations

Although this study revealed a face-specific processing delay in patients with schizophrenia that was correlated with clinical symptoms, the key brain structure corresponding to this delay was not determined. Simultaneous measurement of the ERP and fMRI in future studies may provide more precise temporal dynamics and spatial locus of impaired face-specific processing in schizophrenia. This study only discussed the results relative to the demyelination hypothesis of schizophrenia; advanced MRI technology could be used in future studies to provide a myelin density map for further validation (Glasser et al., 2016).

## Conclusion

This study investigated face-specific neurophysiological markers and their relationships with clinical symptoms in people with schizophrenia and in healthy controls. Compared with healthy controls, significant N170 latency sluggishness was found in the schizophrenia group. This neural timing sluggishness increased with the severity of both negative and general mental symptoms, suggesting a correlation between dysfunctions in face-specific processing and the development of symptoms in people with schizophrenia.

## Author Contributions

YZ, HL, YN, JR, ZW, RH, TL, GL, TB, QW and SS contributed to the design of the study and wrote protocols. The experiments were conducted by YZ, HL and SS. QW wrote the first draft of the manuscript. All authors contributed to and approved the final manuscript.

## Conflict of Interest Statement

The authors declare that the research was conducted in the absence of any commercial or financial relationships that could be construed as a potential conflict of interest.

## Footnotes

^1^www.adobe.com

^2^www.neuro.uestc.edu.cn/rest
