# Supplementary material for: Sluggishness of Early-Stage Face Processing (N170) Is Correlated with Negative and General Psychiatric Symptoms in Schizophrenia
Source: Front Hum Neurosci. 2016 Nov 28;10:615. doi: 10.3389/fnhum.2016.00615 (PMC5124944; doi:10.3389/fnhum.2016.00615)
Supplement: Supplementary file 1 [file DataSheet_1.pdf]

Supplementary Information

Sluggishness of Early-Stage Face Processing (N170) is Correlated with Negative and General Psychiatric Symptoms in Schizophrenia

Yingjun Zheng[1, #], Haijing Li[1, #], Yuping Ning[1], Jianjuan Ren[1], Zhangying Wu[1], Rongcheng Huang[1], Tianfu Li[2,3,4], Guoming Luan[2,3,4], Taiyong Bi[5,6], Qian Wang[2,*], Shenglin She[1,*]

[1]Department of General Psychiatry, The Affiliated Brain Hospital of Guangzhou Medical University (Guangzhou Huiai Hospital), Guangzhou, China;

[2]Beijing Key Lab of Epilepsy, Epilepsy Center, Beijing Sanbo Brain Hospital, Capital Medical University, Beijing, China;

[3]Department of Functional Neurosurgery, Beijing Sanbo Brain Hospital, Capital Medical University, Beijing, China;

[4]Beijing Institute for Brain Disorders, Beijing, China;

[5]Key Laboratory of Cognition and Personality (SWU), Ministry of Education, Chongqing China;

[6]Faculty of Psychology, Southwest University, Chongqing, China

[#]These authors contribute equally in this work.

***Corresponding author:**

Shenglin She

Department of Psychiatry

The Affiliated Brain Hospital of Guangzhou Medical University (Guangzhou Huiai Hospital)

Guangzhou, China, 510370

Tel: +86 020 81268107

Fax: +86 020 81891391

Email: 15013144670@126.com

&

Guoming Luan

Beijing Key Lab of Epilepsy, Epilepsy Center, Beijing Sanbo Brain Hospital, Capital Medical University, Beijing, China, 100093

Tel: +86 010 62856718

Fax: +86 010 62856718

Email: luangm3@163.com

&

Qian Wang

Beijing Key Lab of Epilepsy, Epilepsy Center, Beijing Sanbo Brain Hospital, Capital Medical University, Beijing, China, 100093

Tel: +86 010 62856910

Fax: +86 010 62856910

Email: aleinwangba@126.com

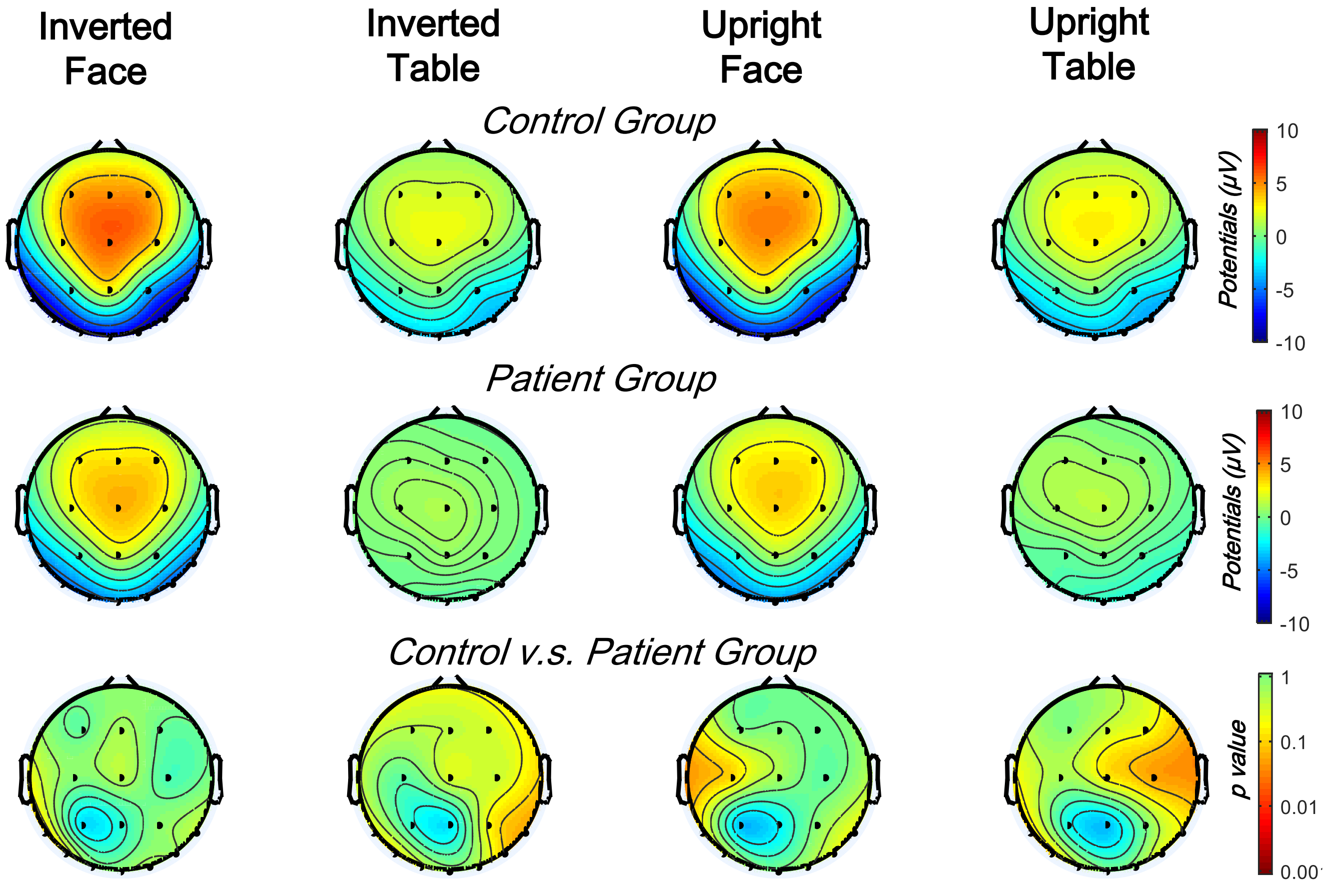

Supplementary Figure S1. The topographic distributions of the N170 mean amplitudes (140-180 ms) under four conditions in control (*top panel*) and patient (*middle panel*) group and between group significance analysis (*bottom panel,* with *Bonferoni* correction). (approximate zero reference, REST)

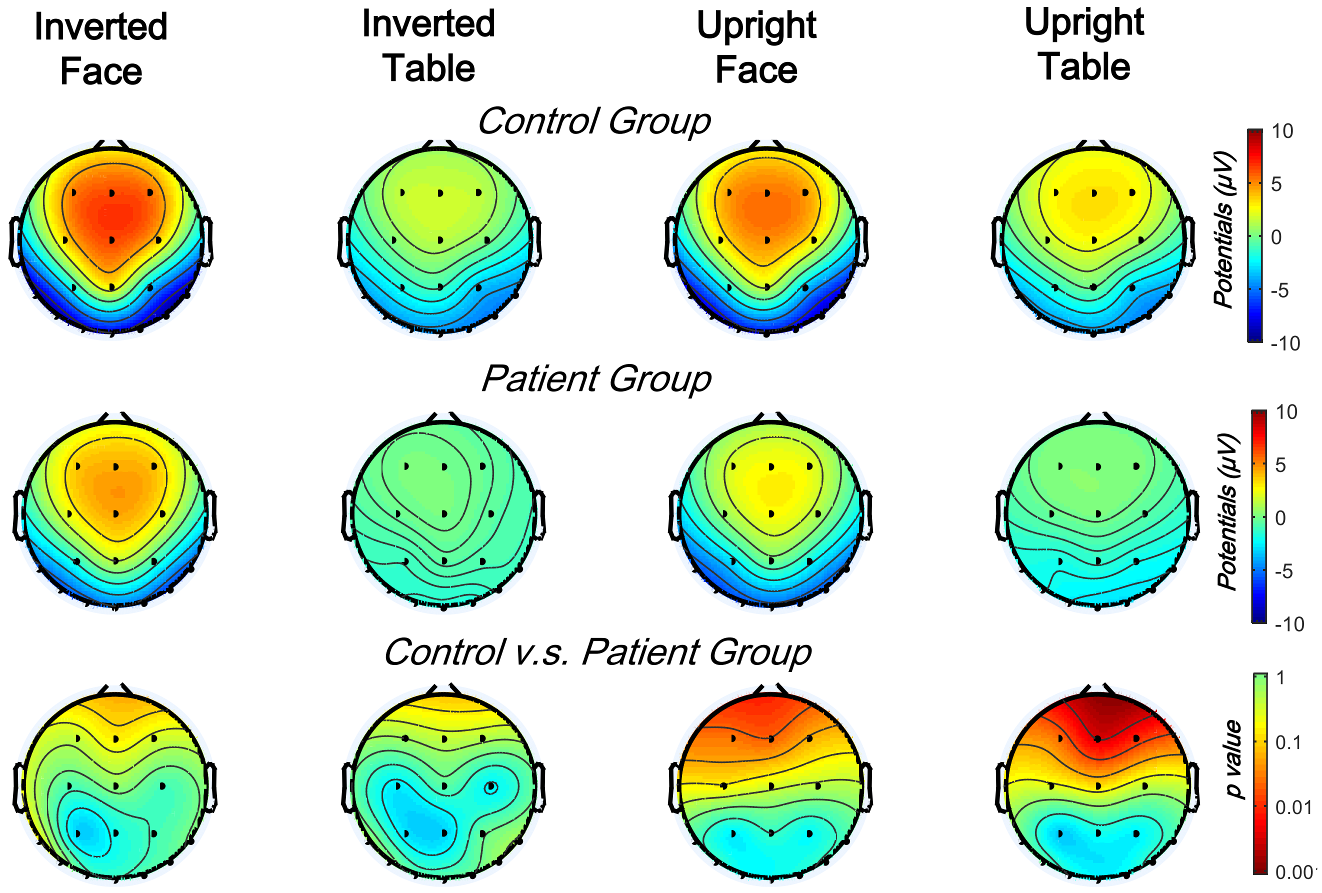

Supplementary Figure S2. The topographic distributions of the N170 mean amplitudes (140-180 ms) under four conditions in control (*top panel*) and patient (*middle panel*) group and between group significance analysis (*bottom panel,* with *Bonferoni* correction). (Average Reference, AR)

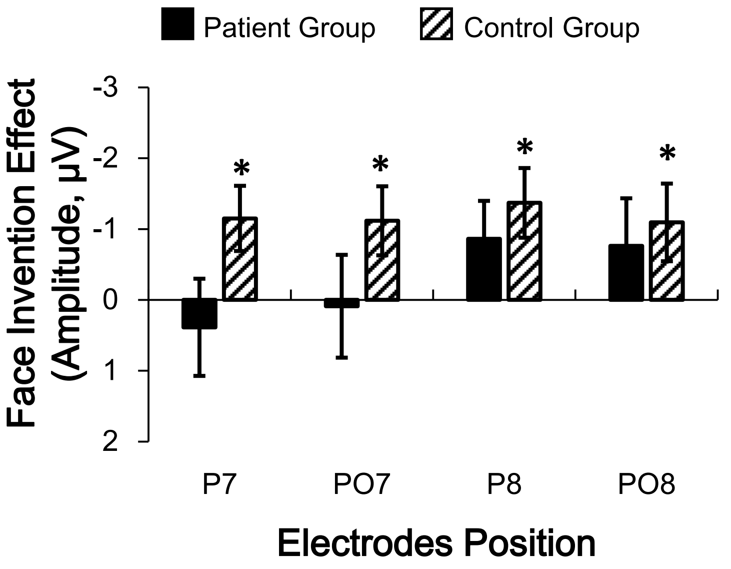

Supplementary Figure S3. Comparison of face inversion effect (FIE) between patient group and control group (P7, P8, PO7, PO8). *Black bars:* schizophrenic patient group; *Striped bars*: healthy control group. *, $p < 0.05$.

Supplementary Table S1. Clinical and demographic characteristics of the patients and healthy controls.

	**Patients with Schizophrenia (n = 24)**	**Healthy Control Subjects (n = 24)**	***P***
**Gender (male/ female)**	12/ 12	12/ 12	1.000 [a]
**Education (years)**	12.9 (2.6)	12.5 (3.5)	0.707 [b]
**Handedness (right/ left)**	24/ 0	24/ 0	
**Schizophrenia Subtypes: Paranoid/ Undifferentiated**	16/ 9	N/ A	
**Duration of illness (years)**	8.7 (6.3)	N/ A	
**PANSS total**	52.4 (12.4)	32.3 (1.5)	0.000 [b]
**PANSS positive symptoms**	13.3 (5.4)	7.4 (0.7)	0.000 [b]
**PANSS negative symptoms**	14.8 (4.7)	7.2 (0.4)	0.000 [b]
**PANSS general symptoms**	27.9 (6.2)	17.7 (1.2)	0.000 [b]
**Antipsychotic meication (Atypical/ Typical)**	22/ 2	N/ A	
**Chlorpromazine equivalent (mg)**	556.5 (350.2)	N/ A	

[a]*Binomial,* [b]*T-test*

Supplementary Table S2. The statistical results of N170 latency (approximate zero reference, REST)

	Main Effects						Interaction Effects							
**Electrodes**	**Face**		**Invert**		**Group**		**Face×Group**		**Invert×Group**		**Face×Invert**		**Face×Invert×Group**	
	F (1,46)	*p*	*F (1,46)*	*p*	*F (1,46)*	*p*	*F (1,46)*	*p*	*F (1,46)*	*p*	*F (1,46)*	*p*	*F (1,46)*	*p*
**O1**	0.572	0.453	0.120	0.731	**6.452***	**0.015**	0.241	0.626	1.172	0.285	2.391	0.129	0.007	0.933
**P3**	0.396	0.546	3.275	0.077	0.002	0.967	0.056	0.813	4.001	0.051	0.001	0.982	0.252	0.618
**P7**	0.426	0.517	**4.257***	**0.045**	**9.992****	**0.003**	0.462	0.500	0.811	0.373	0.306	0.583	0.601	0.442
**PO7**	3.774	0.058	2.846	0.098	**7.358****	**0.009**	0.205	0.653	0.337	0.564	**6.918***	**0.012**	0.523	0.473
**Oz**	1.136	0.292	1.724	0.196	3.410	0.071	0.018	0.895	0.330	0.568	0.180	0.673	2.741	0.105
**PZ**	0.231	0.633	0.002	0.967	0.344	0.561	0.090	0.766	0.009	0.923	1.672	0.202	3.407	0.071
**O2**	1.203	0.278	1.859	0.179	3.188	0.081	0.021	0.885	0.299	0.587	0.095	0.759	2.788	0.102
**P4**	0.503	0.482	0.640	0.428	0.140	0.710	0.723	0.400	0.412	0.524	0.332	0.567	0.656	0.422
**P8**	**10.351****	**0.002**	**5.286***	**0.026**	0.655	0.423	**4.674***	**0.036**	2.605	0.113	0.239	0.628	0.661	0.420
**PO8**	**9.699****	**0.003**	**4.111***	**0.048**	1.537	0.221	3.146	0.083	1.465	0.232	0.550	0.462	0.591	0.446

Supplementary Table S3. The statistical results of N170 latency (average reference, AR)

	Main Effects						Interaction Effects							
**Electrodes**	**Face**		**Invert**		**Group**		**Face×Group**		**Invert×Group**		**Face×Invert**		**Face×Invert×Group**	
	F (1,46)	*p*	*F (1,46)*	*p*	*F (1,46)*	*p*	*F (1,46)*	*p*	*F (1,46)*	*p*	*F (1,46)*	*p*	*F (1,46)*	*p*
**O1**	0.813	0.372	**8.468****	**0.006**	**4.682***	**0.036**	1.756	0.192	3.046	0.088	0.030	0.863	0.504	0.481
**P3**	0.845	0.363	2.767	0.103	0.013	0.910	0.282	0.598	1.777	0.189	1.470	0.232	0.924	0.342
**P7**	3.456	0.069	**5.457***	**0.024**	**5.093***	**0.029**	3.213	0.080	0.797	0.377	0.000	0.995	0.417	0.522
**PO7**	0.767	0.386	**7.810****	**0.008**	**5.811***	**0.020**	1.349	0.251	1.266	0.266	0.304	0.584	0.134	0.716
**Oz**	1.200	0.279	1.251	0.269	2.708	0.107	3.108	0.085	0.159	0.692	0.309	0.581	0.205	0.653
**PZ**	1.012	0.320	0.091	0.764	0.876	0.354	0.035	0.853	0.292	0.592	0.859	0.359	0.927	0.341
**O2**	2.348	0.132	**11.616****	**0.001**	2.180	0.147	3.715	0.060	3.012	0.089	0.000	0.960	0.030	0.850
**P4**	3.309	0.075	2.957	0.092	0.004	0.949	1.417	0.240	2.623	0.112	0.005	0.946	0.003	0.957
**P8**	0.024	0.877	1.409	0.241	2.413	0.127	0.678	0.414	0.006	0.936	0.259	0.613	1.571	0.216
**PO8**	0.909	0.345	0.587	0.447	1.959	0.168	2.612	0.113	0.019	0.891	0.051	0.822	0.360	0.522

Supplementary Table S4. The statistical results of N170 amplitude (approximate zero reference, REST)

	Main Effects						Interaction Effects							
**Electrodes**	**Face**		**Invert**		**Group**		**Face×Group**		**Invert×Group**		**Face×Invert**		**Face×Invert×Group**	
	F (1,46)	*p*	*F (1,46)*	*p*	*F (1,46)*	*p*	*F (1,46)*	*p*	*F (1,46)*	*p*	*F (1,46)*	*p*	*F (1,46)*	*p*
**O1**	**34.526*** **	**0.000**	1.358	0.250	3.829	0.056	0.034	0.854	0.939	0.337	1.130	0.293	0.001	1.000
**P3**	1.744	0.193	0.206	0.652	0.649	0.424	0.083	0.775	1.999	0.164	0.524	0.473	0.026	0.872
**P7**	**57.543** **	**0.001**	0.016	0.910	**6.123* **	**0.017**	2.033	0.161	0.044	0.834	**5.465* **	**0.024**	0.721	0.400
**PO7**	**48.592*** **	**0.000**	0.345	0.555	**5.202* **	**0.027**	0.657	0.422	0.663	0.420	**4.965* **	**0.031**	0.674	0.416
**Oz**	**23.970** **	**0.000**	0.237	0.629	2.797	0.101	0.610	0.439	0.004	0.950	0.482	0.491	0.009	0.926
**PZ**	0.018	0.895	0.024	0.033	0.029	0.865	0.272	0.604	1.283	0.263	0.821	0.370	0.910	0.345
**O2**	**28.666** **	**0.000**	0.017	0.896	3.358	0.073	1.165	0.286	0.075	0.786	0.239	0.627	0.063	0.803
**P4**	4.024	0.051	1.009	0.320	3.725	0.060	1.120	0.296	2.376	0.130	0.089	0.767	0.041	0.841
**P8**	**22.215*** **	**0.000**	0.982	0.327	3.749	0.059	0.399	0.530	0.679	0.414	0.180	0.673	1.682	0.201
**PO8**	**19.580*** **	**0.000**	1.174	0.678	3.242	0.078	0.124	0.727	0.114	0.737	0.020	0.888	2.058	0.158

Supplementary Table S5. The statistical results of N170 amplitude (average reference, AR)

	Main Effects						Interaction Effects							
**Electrodes**	**Face**		**Invert**		**Group**		**Face×Group**		**Invert×Group**		**Face×Invert**		**Face×Invert×Group**	
	F (1,46)	*p*	*F (1,46)*	*p*	*F (1,46)*	*p*	*F (1,46)*	*p*	*F (1,46)*	*p*	*F (1,46)*	*p*	*F (1,46)*	*p*
**O1**	**16.154*** **	**0.000**	3.228	0.079	2.660	0.110	0.107	0.745	3.308	0.075	0.028	0.869	0.003	0.955
**P3**	2.014	0.163	**13.485****	**0.001**	0.215	0.645	0.699	0.408	**11.215****	**0.002**	0.138	0.712	0.001	0.976
**P7**	**11.920****	**0.001**	3.472	0.069	**4.311***	**0.043**	0.157	0.693	**4.546***	**0.038**	0.102	0.751	0.355	0.554
**PO7**	**16.136*****	**0.000**	2.338	0.133	3.649	0.062	0.006	0.938	3.445	0.070	0.006	0.940	0.060	0.808
**Oz**	**12.995****	**0.001**	**5.979***	**0.018**	2.273	0.138	0.002	0.968	**5.123***	**0.028**	0.035	0.852	0.006	0.938
**PZ**	0.271	0.605	**14.842*****	**0.000**	0.008	0.930	0.101	0.752	**12.723****	**0.001**	0.398	0.531	1.286	0.263
**O2**	**11.442****	**0.001**	3.984	0.052	2.615	0.113	0.038	0.845	3.657	0.062	0.237	0.629	0.071	0.792
**P4**	0.303	0.584	**10.416****	**0.002**	1.976	0.166	0.002	0.964	**8.336****	**0.006**	0.801	0.376	0.930	0.340
**P8**	**15.202*****	**0.000**	2.554	0.117	2.777	0.102	0.040	0.843	3.644	0.063	3.069	0.086	0.884	0.352
**PO8**	**16.452*****	**0.000**	1.843	0.181	2.437	0.125	0.956	0.742	2.257	0.140	2.545	0.117	0.770	0.385
